# Rutin administration attenuates myocardial dysfunction in diabetic rats

**DOI:** 10.1186/s12933-015-0255-7

**Published:** 2015-07-17

**Authors:** Julliano F C Guimaraes, Bruno P Muzio, Camila M Rosa, Andre F Nascimento, Mario M Sugizaki, Ana A H Fernandes, Antonio C Cicogna, Carlos R Padovani, Marina P Okoshi, Katashi Okoshi

**Affiliations:** Department of Internal Medicine, Botucatu Medical School, Sao Paulo State University, UNESP, Botucatu, Brazil; Department of Chemistry and Biochemistry, Institute of Biosciences, Sao Paulo State University, UNESP, Botucatu, Brazil; Departamento de Clinica Medica, Faculdade de Medicina de Botucatu, UNESP Rubiao Junior, S/N 18618-970, Botucatu, SP Brazil

**Keywords:** Diabetes mellitus, Anti-oxidant, Cardiac remodeling, Papillary muscle, Rat, Flavonoid

## Abstract

**Background:**

Oxidative stress plays a major role in diabetic cardiomyopathy pathogenesis. Anti-oxidant therapy has been investigated in preventing or treating several diabetic complications. However, anti-oxidant action on diabetic-induced cardiac remodeling is not completely clear. This study evaluated the effects of rutin, a flavonoid, on cardiac and myocardial function in diabetic rats.

**Methods:**

Wistar rats were assigned into control (C, n = 14); control-rutin (C-R, n = 14); diabetes mellitus (DM, n = 16); and DM-rutin (DM-R, n = 16) groups. Seven days after inducing diabetes (streptozotocin, 60 mg/kg, i.p.), rutin was injected intraperitoneally once a week (50 mg/kg) for 7 weeks. Echocardiogram was performed and myocardial function assessed in left ventricular (LV) papillary muscles. Serum insulin concentration was measured by ELISA. Statistics: One-way ANOVA and Tukey’s post hoc test.

**Results:**

Glycemia was higher in DM than DM-R and C and in DM-R than C-R. Insulin concentration was lower in diabetic groups than controls (C 2.45 ± 0.67; C-R 2.09 ± 0.52; DM 0.59 ± 0.18; DM-R 0.82 ± 0.21 ng/mL). Echocardiogram showed no differences between C-R and C. DM had increased LV systolic diameter compared to C, and increased left atrium diameter/body weight (BW) ratio and LV mass/BW ratio compared to C and DM-R. Septal wall thickness, LV diastolic diameter/BW ratio, and relative wall thickness were lower in DM-R than DM. Fractional shortening and posterior wall shortening velocity were lower in DM than C and DM-R. In papillary muscle preparation, DM and DM-R presented higher time to peak tension and time from peak tension to 50% relaxation than controls; time to peak tension was lower in DM-R than DM. Under 0.625 and 1.25 mM extracellular calcium concentrations, DM had higher developed tension than C.

**Conclusion:**

Rutin attenuates cardiac remodeling and left ventricular and myocardial dysfunction caused by streptozotocin-induced diabetes mellitus.

## Background

Diabetes mellitus is an important public health problem because of a high prevalence and increased morbidity and mortality. Cardiovascular involvement is frequent in diabetes and a major cause of death in diabetic patients [1, 2]. Cardiac alterations are caused by both coronary atherosclerosis and diabetes-related cardiomyopathy. As first reported by Rubler et al. [3], diabetic cardiomyopathy is characterized by left ventricular systolic and diastolic dysfunction in the absence of underlying coronary artery disease and/or systemic arterial hypertension [4]. Diabetic cardiomyopathy may affect both type 1 and 2 diabetic patients [5]. The pathophysiology of diabetic cardiomyopathy is multifactorial and includes several mechanisms such as myocardial fibrosis, myocyte hypertrophy, contractile dysfunction, changes in calcium handling and mitochondrial function, impaired nitric oxide signaling, and abnormal cardiomyocyte loss by apoptosis [5–7]. Despite all these mechanisms, oxidative stress is widely considered a major cause in the pathogenesis of diabetic cardiomyopathy [8–10].

In diabetes, oxidative stress can be induced by hyperglycemia, hyperlipidemia, and inflammation. Oxidative stress occurs when the production of reactive oxygen species exceeds their degradation by anti-oxidant defenses [9, 11]. Anti-oxidant therapy has been extensively studied to prevent or treat several diabetic complications [12–14]; however, direct investigations on the role of anti-oxidants on diabetic-induced cardiac remodeling remain relatively scarce [9].

Flavonoids are natural substances with variable phenolic structures that have been used for their anti-oxidant properties [15]. In diabetic rats, flavonoids reversed cardiac dysfunction and structural changes and attenuated oxidative stress and cardiac dysfunction induced by ischemia–reperfusion injury [16, 17]. More than 4,000 varieties of flavonoids have been identified [15, 18]. One of the main flavonoids is rutin, which can be extracted from many nature sources, including buckwheat, oranges, grapes, lemons, limes, peaches, and berries [19]. Recently, rutin was shown to attenuate oxidative stress, apoptosis, and inflammation in diabetic rat hearts [19]. In this study, we evaluated the effects of rutin on cardiac and myocardial function in rats with streptozotocin-induced diabetes.

## Methods

Male Wistar rats weighing 300–350 g were purchased from the Central Animal House at Botucatu Medical School, UNESP. All animals were housed in a temperature controlled room 25 ± 2ºC and kept on a 12 h light/dark cycle. All experiments and procedures were approved by Botucatu Medical School Ethics Committee, Sao Paulo State University, UNESP, and performed in compliance with the ARRIVE guidelines on animal research [20].

Diabetes was induced by intraperitoneal injection of streptozotocin (STZ, Sigma, St. Louis, MO, USA) at a dose of 60 mg/kg body weight, dissolved in 0.1 M citrate buffer, pH 4.5 [21, 22]. Forty-eight hours after STZ administration, blood glucose was measured by glucometer (Boehringer Mannheim, Eli Lilly Ltd., São Paulo, Brazil). Only STZ-treated rats with glycemia >250 mg/dL were considered diabetic and included in the study [23]. Rats were randomly assigned into four groups:

1. control group (C, n = 14)—rats treated with vehicle;
2. control-rutin (C-R, n = 14) group—rats treated with rutin;
3. diabetes mellitus (DM, n = 16) group—diabetic rats treated with vehicle;
4. DM-rutin (DM-R, n = 16) group—diabetic rats treated with rutin.

Seven days after STZ injection, rutin (Sigma, St. Louis, MO, USA) was administered once a week at a dose of 50 mg/kg body weight, for 7 weeks. Rutin was solubilized in 500 μL of propylene glycol; the volume was then completed to 500 μL with water and injected intraperitoneally. Food and water were supplied ad libitum. To adjust rutin dose, individual rat food (g) and water (mL) intake was measured daily. Body weight was measured weekly. At the end of experimental period (56 days), rats were fasted for 12 h, anesthetized, and decapitated.

### Echocardiographic study

Echocardiographic evaluation was performed at the end of the study using a commercially available echocardiograph (General Electric Medical Systems, Vivid S6, Tirat Carmel, Israel) equipped with a 5–11.5 MHz multifrequency probe, as previously described [24–26]. Rats were anesthetized by intramuscular injection of a ketamine (50 mg/kg) and xylazine (0.5 mg/kg) mixture. A two-dimensional parasternal short-axis view of the left ventricle (LV) was obtained at the level of the papillary muscles. M-mode tracings were obtained from short-axis views of the LV at or just below the tip of the mitral-valve leaflets, and at the level of the aortic valve and left atrium. M-mode images of the LV were printed on a black-and-white thermal printer (Sony UP-890MD) at a sweep speed of 100 mm/s. All LV structures were manually measured by the same observer (KO) according to the leading-edge method of the American Society of Echocardiography. Means were obtained from measurements of at least five cardiac cycles on the M-mode tracings. The following structural variables were measured: LV diastolic and systolic diameters (LVDD and LVSD, respectively), LV diastolic posterior wall thickness (PWT), LV diastolic septal wall thickness (SWT), and left atrium diameter (LA). Left ventricular mass (LVM) was calculated using the formula [(LVDD + PWT + SWT)^3^ − (LVDD)^3^] × 1.04. LV relative wall thickness (RWT) was calculated by the formula 2 × PWT/LVDD. LV function was assessed by the following parameters: endocardial fractional shortening (FS), posterior wall shortening velocity (PWSV), early and late diastolic mitral inflow velocities (E and A waves), and E/A ratio [27].

### Myocardial functional study

One day after the echocardiographic study, myocardial intrinsic contractile function was evaluated in isolated LV papillary muscles as previously described [28–30]. Rats were anesthetized (pentobarbital sodium, 50 mg/kg, intraperitoneally) and decapitated. Hearts were quickly removed and placed in oxygenated Krebs–Henseleit solution at 28°C. LV anterior or posterior papillary muscle was dissected free, mounted between two spring clips, and placed vertically in a chamber containing Krebs–Henseleit solution at 28°C and oxygenated with a mixture of 95% O_2_ and 5% CO_2_ (pH 7.38). Krebs–Henseleit solution composition in mM was as follows: 118.5 NaCl, 4.69 KCl, 2.50 CaCl_2_, 1.16 MgSO_4_, 1.18 KH_2_PO_4_, 5.50 glucose, and 25.88 NaHCO_3_. The spring clips were attached to a Kyowa model 120T-20B force transducer and a lever system, which allowed for muscle length adjustment. Preparations were stimulated 12 times/min at a voltage 10% above threshold.

After a 60 min period, during which preparations were permitted to shorten while carrying light loads, muscles were loaded to isometrically contract and stretched to the apices of their length-tension curves. After a 5 min period, during which preparations performed isotonic contractions, muscles were again placed under isometric conditions, and the apex of the length-tension curve (L_max_) was determined. A 15 min period of stable isometric contraction was imposed prior to the experimental period. One isometric contraction was then recorded for later analysis.

The following parameters were measured from isometric contraction: peak of developed tension (DT, g/mm^2^), time to peak of tension (TPT, ms), time from peak tension to 50% relaxation (RT_1/2_), maximum rate of tension development (+dT/dt, g/mm^2^/s), and maximum rate of tension decline (−dT/dt, g/mm^2^/s). Myocardial function was also evaluated under different extracellular Ca^2+^ concentrations [31, 32].

Papillary muscle cross-sectional area was calculated from muscle weight and length by assuming cylindrical uniformity and a specific gravity of 1.0. All force data were normalized for muscle cross-sectional area [33].

### Anatomical parameters

After dissecting papillary muscle, right and left ventricles were separated and weighed. Lung fragments were weighed before and after drying sessions (65°C for 72 h) to evaluate wet/dry weight ratio [34, 35].

### Biochemical analyses

Glycemia was measured by the enzymatic method using glucose oxidase and peroxidase. Serum insulin concentration was determined by enzyme immune assay kit (EIA kit, Cayman Chemical, USA), using an ELISA reader (Biotech Instruments, Inc., USA) [36].

### Statistical analysis

Results are expressed as mean ± standard deviation. Statistical differences between groups were assessed by analysis of variance (ANOVA) in a factorial design 2 × 2 in the one-way model. The Tukey’s post hoc test was used to compare individual groups, when the ANOVA revealed a significant F statistic. Statistical significance was accepted at p < 0.05. The statistical analyses were performed using Systat for Windows version 12.0 (Systat Software Inc, San Jose, CA, USA).

## Results

None of the rats died during the experimental period. Forty-eight hours after streptozotocin administration, glycemia was significantly higher in diabetic than control groups (C 93.1 ± 3.0; C-R 84.9 ± 6.0; DM 414 ± 77; DM-R 417 ± 78 mg/dL). At the end of the experiment, glycemia was statistically higher in DM than DM-R and C and in DM-R than C-R (C 98.9 ± 13.7; C-R 104 ± 11.9; DM 397 ± 57.5; DM-R 149 ± 30.3 mg/dL). At the end of the study, serum insulin concentration was significantly lower in both diabetic groups compared to their respective controls (C 2.45 ± 0.67; C-R 2.09 ± 0.52; DM 0.59 ± 0.18; DM-R 0.82 ± 0.21 ng/mL).

Table 1 shows anatomical parameters. At the end of experiment, body weight (BW) was lower in DM and DM-R than their respective controls and higher in DM-R than DM. LV and right ventricle weight was lower in DM and DM-R than controls.

**Table 1:** Anatomical parameters

Parameters	C (n = 14)	C-R (n = 14)	DM (n = 16)	DM-R (n = 16)
BW Initial (g)	365 ± 24	360 ± 24	409 ± 34	370 ± 27
BW Final (g)	444 ± 42	434 ± 58	287 ± 33*	345 ± 62^#§^
LV weight (g)	0.85 ± 0.09	0.77 ± 0.13	0.58 ± 0.12*	0.68 ± 0.10^#^
RV weight (g)	0.25 ± 0.04	0.21 ± 0.05*	0.17 ± 0.05*	0.17 ± 0.05^#^
Wet/dry Lung	4.72 ± 0.76	4.72 ± 0.92	4.84 ± 0.21	4.98 ± 0.55

Data are expressed as mean ± standard deviation or median and percentiles 25 and 75%. One-way ANOVA in a factorial design 2 × 2 and Tukey’s post hoc test.

*C* untreated rats, *C-R* rutin-treated rats, *DM* untreated diabetic rats, *DM-R* rutin-treated diabetic rats, *BW* body weight, *LV* left ventricle, *RV* right ventricle, *Wet/dry* wet-to-dry weight ratio.

* p < 0.05 vs C; ^#^p < 0.05 vs C-R; ^§^p < 0.05 vs DM.

Table 2 shows cardiac structural echocardiographic parameters evaluated at the end of the experimental period. C-R did not differ from C. Both DM and DM-R presented decreased septal wall thickness and relative wall thickness, and increased LV diastolic diameter/BW ratio than their respective controls. DM had increased LV systolic diameter compared to C, and increased left atrium diameter/BW ratio, and LV mass/BW ratio than both C and DM-R groups. DM-R presented lower LV posterior wall thickness, left atrium diameter, and LV mass than C-R, and lower septal wall thickness, LV diastolic diameter/BW ratio, and relative wall thickness than DM. Table 3 shows LV function. C-R did not differ from C. DM had reduced fractional shortening and posterior wall shortening velocity than C and DM-R. DM-R presented higher A wave and lower E/A ratio than DM.

**Table 2:** Echocardiographic structural data

Parameters	C (n = 14)	C-R (n = 14)	DM (n = 16)	DM-R (n = 16)
HR (bpm)	292 ± 33	293 ± 32	271 ± 57	283 ± 30
LVDD (mm)	8.12 ± 0.42	8.21 ± 0.36	8.30 ± 0.46	8.35 ± 0.51
LVSD (mm)	3.75 ± 0.57	3.91 ± 0.34	4.26 ± 0.36*	3.97 ± 0.50
PWT (mm)	1.53 ± 0.11	1.53 ± 0.10	1.46 ± 0.07	1.34 ± 0.06^#^
SWT (mm)	1.65 ± 0.05	1.63 ± 0.08	1.48 ± 0.10*	1.40 ± 0.07^#§^
LA (mm)	5.76 ± 0.64	6.03 ± 0.71	5.73 ± 0.56	5.36 ± 0.53^#^
LVDD/BW (mm/kg)	18.4 ± 1.81	19.2 ± 2.11	29.2 ± 3.07*	24.7 ± 3.26^#§^
LA/BW (mm/kg)	13.0 ± 1.59	14.1 ± 2.47	20.1 ± 2.09*	16.0 ± 2.85^§^
LVM (g)	0.94 ± 0.10	0.95 ± 0.10	0.89 ± 0.12	0.81 ± 0.11^#^
LVM/BW (g/kg)	2.13 ± 0.25	2.23 ± 0.30	3.12 ± 0.47*	2.38 ± 0.23^§^
RWT	0.38 ± 0.04	0.38 ± 0.04	0.36 ± 0.02*	0.32 ± 0.02^#§^

Data are expressed as mean ± standard deviation. One-way ANOVA in a factorial design 2 × 2 and Tukey’s post hoc test.

*C* untreated rats, *C-R* rutin-treated rats, *DM* untreated diabetic rats, *DM-R* rutin-treated diabetic rats, *HR* heart rate, *LVDD and LVSD* left ventricular (LV) diastolic and systolic diameter, respectively, *PWT* LV posterior wall thickness, *SWT* septal wall thickness, *LA* left atrial diameter, *LVM* LV mass, *RWT* relative wall thickness, *BW* body weight.

* p < 0.05 vs C; ^#^p < 0.05 vs C-R; ^§^ < 0.05 vs DM.

**Table 3:** Echocardiographic left ventricular functional data

Parameters	C (n = 14)	C-R (n = 14)	DM (n = 16)	DM-R (n = 16)
FS (%)	54.1 ± 5.2	52.5 ± 2.87	48.7 ± 3.4*	52.6 ± 4.2^§^
PWSV (mm/s)	44.5 ± 4.6	44.3 ± 3.54	35.5 ± 5.4*	42.7 ± 4.6^§^
E (cm/s)	74.1 ± 11.7	66.6 ± 10.2	75.1 ± 14.3	79.8 ± 12.2^#^
A (cm/s)	52.9 ± 11.3	45.5 ± 8.82	48.1 ± 18.1	63.1 ± 21.8^#§^
E/A	1.43 ± 0.23	1.51 ± 0.31	1.77 ± 0.82	1.36 ± 0.34^§^

Data are expressed as mean ± standard deviation. One-way ANOVA in a factorial design 2 × 2 and Tukey’s post hoc test.

*C* untreated rats, *C-R* rutin-treated rats, *DM* untreated diabetic rats, *DM-R* rutin-treated diabetic rats, *FS* endocardial fractional shortening, *PWSV* posterior wall shortening velocity, *E* early diastolic mitral inflow velocity, *A* late diastolic mitral inflow velocity, *E/A* E/A ratio.

* p < 0.05 vs C; ^#^p < 0.05 vs C-R; ^§^p < 0.05 vs DM.

**Figure 1:**
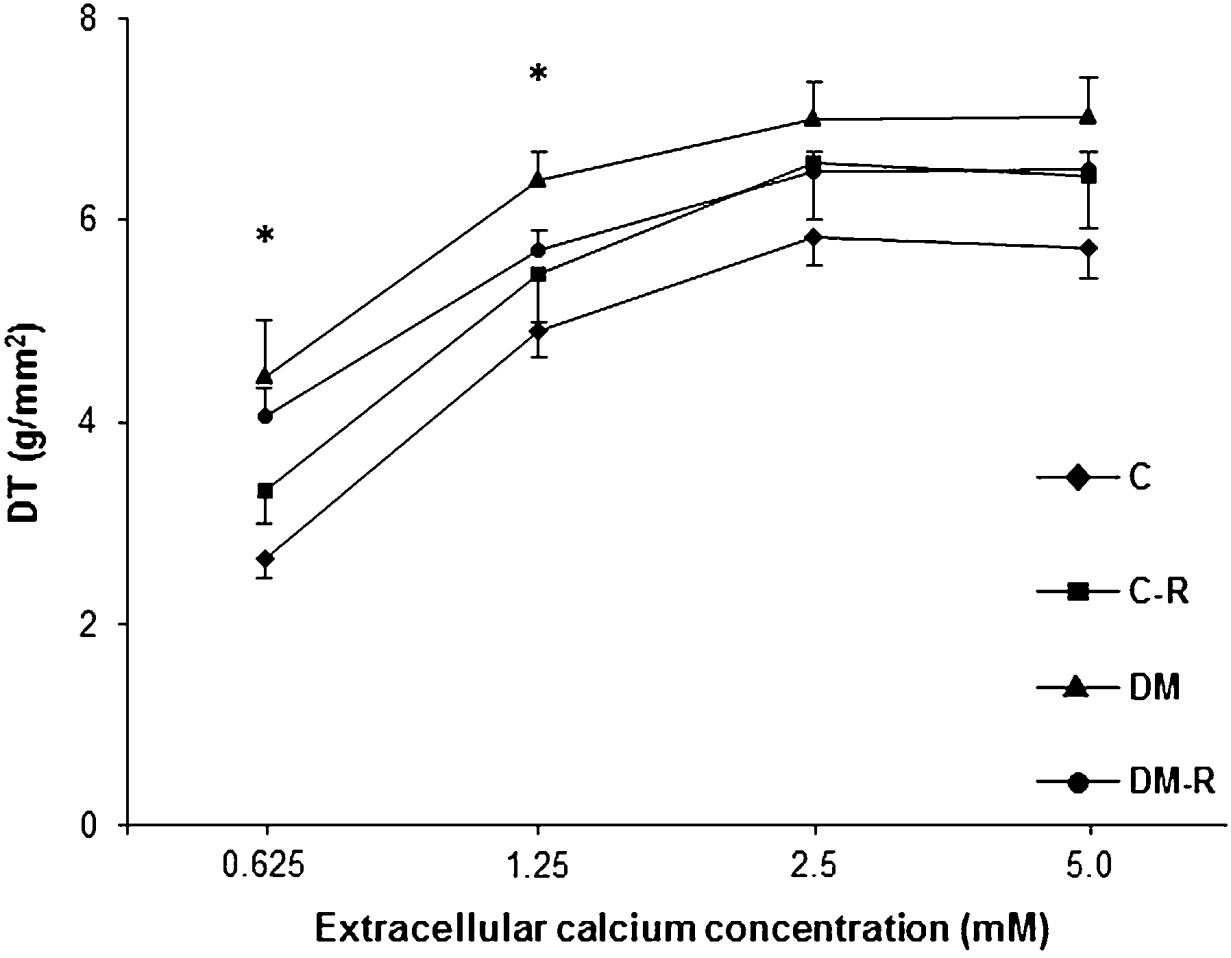
Peak of developed tension (DT) under different extracellular calcium concentrations. *C* untreated rats, *C-R* rutin-treated rats, *DM* untreated diabetic rats, *DM-R* rutin-treated diabetic rats. Values are mean and standard error; Anova and Tukey; *p < 0.05 DM vs C.

Table 4 shows basal papillary muscle functional data. DM and DM-R presented higher time to peak of tension and time from peak tension to 50% relaxation than their respective controls. DM-R had a lower time to peak of tension than DM. Under 0.625 and 1.25 mM extracellular calcium concentrations, DM had higher developed tension than C (Figure 1).

**Table 4:** Isolated papillary muscle data at 2.50 mM extracellular Ca^2+^ concentration

Parameters	C (n = 10)	C-R (n = 13)	DM (n = 13)	DM-R (n = 12)
DT (g/mm^2^)	5.83 ± 0.86	6.57 ± 2.02	7.00 ± 0.85	6.49 ± 0.66
TPT (ms)	148.23 ± 7.45	146.92 ± 8.07	195.12 ± 8.07*	168.33 ± 14.39^#§^
RT_1/2_	159.86 ± 29.59	169.76 ± 14.17	224.62 ± 5.27*	213.43 ± 26.45^#^
+dT/dt (g/mm^2^/s)	59.54 ± 9.53	72.65 ± 21.22	66.65 ± 7.71	69.07 ± 11.14
−dT/dt (g/mm^2^/s)	25.53 ± 4.75	26.93 ± 7.02	23.28 ± 4.25	21.98 ± 3.16
CSA	0.961 ± 0.194	0.962 ± 0.294	0.889 ± 0.140	0.951 ± 0.144

Data are expressed as mean ± standard deviation. One-way ANOVA in a factorial design 2 × 2 and Tukey’s post hoc test.

*C* untreated rats, *C-R* rutin-treated rats, *DM* untreated diabetic rats, *DM-R* rutin-treated diabetic rats, *DT* peak of developed tension, *TPT* time to peak of tension, *RT*_*1/2*_ time from peak tension to 50% relaxation, *+dT/dt* maximum rate of tension development, *−dT/dt* maximum rate of tension decline, *CSA* papillary muscle cross-sectional area.

* p < 0.05 vs C; ^#^p < 0.05 vs C-R; ^§^p < 0.05 vs DM.

## Discussion

In this study we showed that rutin attenuated cardiac structural changes and left ventricular and myocardial dysfunction in rats with streptozotocin-induced diabetes mellitus.

Because of its toxic effect on pancreatic β-cells in the islets of Langerhans, streptozotocin has been widely used to induce type 1 diabetes mellitus in rodents [37–39]. As previously reported [37], streptozotocin-induced diabetes mellitus was characterized by body weight loss and increased blood glucose. Despite similar blood glucose levels 48 h after streptozotocin administration, rutin-treated rats presented attenuation in increased final blood glucose concentration and body weight loss. Administration of isoflavones or isolated rutin has been shown to exert an antihyperglycemic effect in humans with type 2 diabetes mellitus [40] and in experimental models such as streptozotocin-induced diabetes [41, 42] and obese Zucker rats [43]. At the end of our experiment, insulin levels did not statistically differ between DM-R and DM groups. A strong relationship was observed between flavonoids and glucose metabolism. In cell culture, flavonoid procyanidins had insulin-like effects on insulin sensitive cells [41]. This flavonoid effect could explain our result of reduced blood glucose levels despite unchanged insulin levels. Furthermore, flavonoids have been shown to depress gluconeogenic enzymes and inhibit glucose-6-phosphatase in the liver, consequently reducing blood glucose release [44].

Echocardiographic evaluation showed diabetic rats presenting left cardiac chambers dilation with increased LV mass and reduced relative wall thickness, which establishes a pattern of LV eccentric hypertrophy. Some authors did not observe statistically significant changes in echocardiography structures 8 weeks after inducing diabetes in rats [45]. It is probable that, in our study, by using a greater sample size, we could observe significant dilation in left cardiac chambers. Functionally, DM had systolic dysfunction characterized by decreased endocardial fractional shortening and posterior wall shortening velocity.

Several pathophysiological mechanisms can be involved in cardiac injury during diabetes [23, 46]. Impaired myocardial contractility is a major cause of cardiac dilation and dysfunction [47]. We therefore evaluated myocardial function in LV papillary muscle preparations. Diabetes increased contraction and relaxation time. Under low extracellular calcium concentrations, DM had higher developed tension than C, which suggests that diabetes increased myocardial sensitivity to calcium. Alterations in contractile and relaxation function have been previously reported in rats with streptozotocin-induced diabetes [23, 48, 49]. Data on myofibrils sensitivity to calcium are controversial in diabetes [50–53]. Our results are in accordance with some experimental studies. Electrophysiological analysis in rats with alloxan-induced diabetes showed increased muscle sensitivity to a decrease in external calcium [51]. Also skinned fibers from diabetic rats showed a slight increase in calcium sensitivity [54]. This increased calcium sensitivity may have played a role in the slow relaxation time-course characterized by increased time from peak tension to 50% relaxation in DM. Other factors such as lower myosin ATPase activity and abnormal myosin isoenzyme distribution may have contributed to the increased time to peak of tension observed in the DM group [55].

Cardiac parameters in the normoglycemic rats were not changed by rutin, except for reduced right ventricle weight. In the diabetic rats, rutin prevented LV hypertrophy, left atrium dilation, and systolic dysfunction, attenuated LV dilation, and decreased relative wall thickness, thus characterizing cardiac changes as LV eccentric remodeling with preserved systolic function. Myocardial dysfunction was also attenuated by rutin, as time to peak tension was lower in DM-R than DM. Under lower extracellular calcium concentrations, DM-R presented developed tension values between those of C-R and DM and did not significantly differ from either group. Therefore, rutin attenuated cardiac remodeling and myocardial dysfunction in rats with streptozotocin-induced diabetes.

Several beneficial effects of rutin have been shown in different cardiac injury models. In rats fed a high carbohydrate and fat diet, rutin attenuated metabolic changes and cardiac remodeling [56]. In control and diabetic rats, rutin had a cardioprotective role against ischaemia–reperfusion injury [16, 57]. In a clinical setting, this flavonoid decreased systemic blood pressure in diabetic patients [40]. The beneficial effects of flavonoids have been associated with different mechanisms including antiinflammatory, antiallergic, antiviral, and anticarcinogenic properties [15]. However, one of their most important actions is the anti-oxidant effect [15, 58]. Flavonoids stabilize reactive oxygen species by reacting with the reactive compound of the radical. Rutin can inhibit activity of xanthine oxidase, interfere with inducible nitric-oxide synthase activity, inhibit lipoperoxidation and advanced glycation end product generation, and protect against mitochondrial damage and iron chelation [15, 42, 59–61]. In neonatal rat heart myocyte culture, rutin also increased superoxide dismutase activity [57]. Several studies have shown the anti-oxidant potential of flavonoids in diabetes and other diseases [19, 42, 62]. In control and diabetic rats, rutin attenuated oxidative stress and increased antioxidant reserves during ischaemia–reperfusion injury [16, 57]. As oxidative stress plays an important role in the pathophysiology of diabetic cardiomyopathy [22, 23], this study allows us to propose the hypothesis that rutin attenuated diabetes-induced cardiac remodeling and dysfunction through its anti-oxidant properties. Additional studies are needed to clarify the mechanisms of rutin-induced beneficial effects on diabetic cardiomyopathy.

In conclusion, rutin attenuates cardiac remodeling and left ventricular and myocardial dysfunction caused by streptozotocin-induced diabetes mellitus.

## Abbreviations

ANOVA: analysis of variance
A wave: late diastolic mitral inflow velocity
BW: body weight
C: control group
C-R: control-rutin group
CSA: papillary muscle cross-sectional area
DM: diabetes mellitus group
DM-R: DM-rutin group
DT: peak of developed tension
−dT/dt: maximum rate of tension decline
+dT/dt: maximum rate of tension development
E/A: E/A ratio
E wave: early diastolic mitral inflow velocity
FS: endocardial fractional shortening
HR: heart rate
KO: Katashi Okoshi
LA: left atrium diameter
LV: left ventricular
LVDD: left ventricular diastolic diameter
LVM: left ventricular mass
LVSD: left ventricular systolic diameter
PWSV: posterior wall shortening velocity
PWT: left ventricular diastolic posterior wall thickness
RT_1/2_: time from peak tension to 50% relaxation
RWT: left ventricular relative wall thickness
STZ: streptozotocin
SWT: left ventricular diastolic septal wall thickness
TPT: time to peak of tension
